# Stretchable and Wearable Sensors for Contact Touch and Gesture Recognition Based on Poling-Free Piezoelectric Polyester Elastomer

**DOI:** 10.3390/polym17081105

**Published:** 2025-04-19

**Authors:** Kaituo Wu, Wanli Zhang, Qian Zhang, Xiaoran Hu

**Affiliations:** 1 State Key Laboratory of Electronic Thin Films and Integrated Devices, University of Electronic Science and Technology of China, Chengdu 611731, China; 2 School of Materials and Energy, University of Electronic Science and Technology of China, Chengdu 611731, China

**Keywords:** piezoelectricity, triboelectricity, touch control, gesture recognition

## Abstract

Human–computer interaction (HCI) enables communication between humans and computers, which is widely applied in various fields such as consumer electronics, education, medical rehabilitation, and industrial control. Human motion monitoring is one of the most important methods of achieving HCI. In the present work, a novel human motion monitoring sensor for contact touch and gesture recognition is fabricated based on polyester elastomer (PTE) synthesized from diols and diacids, with both piezoelectric and triboelectric properties. The PTE sensor can respond to contacted and contactless me-chemical signals by piezoelectric and triboelectric responding, respectively, which enables simultaneous touch control and gesture recognition. In addition, the PTE sensor presents high stretchability with elongation at break over 1000% and high durability over 4000 impact cycles, offering significant potential for consumer electronics and wearable devices.

## 1. Introduction

Human–computer interaction (HCI) enables interaction between human users and computers or other operating systems. It is currently widely applied in fields such as consumer electronics, education and training, medical rehabilitation, and industrial control [1,2,3,4]. In the field of wearable electronics, traditional HCI technologies mainly rely on input devices such as buttons or keyboard for control, but these methods are increasingly inadequate for complex operating systems. In recent years, emerging HCI technologies such as touch control, gesture recognition, voice interaction, eye tracking, and brain–computer interfaces have attracted significant attention [5,6,7,8]. Among these, gesture recognition, due to its ease of use and precise control advantages, has become the most mature and widely used form of human–computer interaction.

Currently, gesture recognition technology is primarily achieved through external mechanical systems, cameras, infrared sensors, and millimeter-wave sensors [9]. External mechanical systems detect motion and provide feedback to users by using additional sensors, but this can affect the portability of mobile devices and reduce the user experience [10]. Camera-based interactive systems are usually limited by their field of view and shape, making it difficult to detect close motion. Additionally, cameras are highly dependent on environmental factors, such as color, angle, and gesture speed, which can affect their recognition accuracy [11,12,13,14]. Infrared sensors, although capable of detecting body movement, have the disadvantage of large emitter and receiver sizes and high power consumption [15,16]. Millimeter-wave-based sensors can achieve high recognition accuracy, but millimeter waves suffer from significant attenuation in air, which limits their recognition range [17]. Furthermore, gesture recognition often requires the integration of touch control to improve recognition accuracy, but the aforementioned devices are all single components and are difficult to integrate into touch control sensors. The integration of multiple single sensors leads to increasing sensor sizes and power consumption, which goes against the trend of low-power and ultra-lightweight wearable devices. Therefore, researchers aim to develop the next generation of gesture recognition sensors with high integration, flexibility, lightness, and low power consumption.

To meet these demands, piezoelectric and triboelectric polymers show promise. Triboelectric polymers can detect non-contact mechanical signals through electron transfer that occurs when the material suffers contact and separation, while piezoelectric polymers can detect contact mechanical signals by utilizing the induced charge generated when their internal polar crystals deform under external force. Although piezoresistive sensors are sensitive to static mechanical stimuli, piezoelectric sensors are sensitive to dynamical stress, which is more suitable for the touch control function [18,19]. For example, Marchiori et al. [20] developed a stretchable electronic skin with temperature and stress sensing functions using PVDF-TrFE (Polyvinylidene Fluoride-Trifluoroethylene) as the piezoelectric material. Pullano et al. [21] combined PVDF films with infrared sources to develop a contact sensor for rapidly monitoring the rate of biological fluid flow. Therefore, by developing materials with both piezoelectric and triboelectric properties, it could be possible to develop integrated devices for both touch control and gesture recognition.

In this work, a polyester elastomer (PTE) with both piezoelectric and triboelectric properties was synthesized by the copolymerization of several diols and diacids. The long carbon chains in its backbone provide molecular mobility, while the random copolymerization methods and crosslinking sites suppress its crystallization, ensuring PTE with stretchability. The large number of C=O dipoles in the ester bonds enables good piezoelectric and triboelectric properties without polarization. Therefore, the stretchable sensor based on PTE can simultaneously achieve touch control and gesture recognition functions, showing great potential in consumer electronics and wearable devices.

## 2. Experimental Section

### 2.1. Materials

1,3-propanediol (PDO), 2,3-butenediol (BDO), Succinic acid (SuA), sebacic acid (SeA), and itaconic acid (IA) were purchased from Suzhou Suzhen Bioengineering Co., Ltd. (Suzhou, China). Tetrabutyl titanate (TBT) and dicumyl peroxide (DCP) were sourced from Alfa Aesar (Tianjing, China), while the silver paste was supplied by Zhimk Technology Co., Ltd. (Suzhou, China).

### 2.2. Methods

The PTE was synthesized through a two-step process consisting of esterification and polycondensation using monomers including PDO, BDO, SuA, SeA, and IA. The molar ratio of diols to diacids was maintained at 1.1:1, and IA was fixed at 5 mol% to the total reactants. Typically, PDO, BDO, SuA, SeA, and IA were added sequentially to a three-necked flask. The mixture was mechanically stirred at 180 °C under a nitrogen atmosphere for 2 h. Then, 0.1 wt% of tetrabutyl titanate (TBT) was added as a catalyst, and the polycondensation process continued at 180 °C under reduced pressure (≤300 Pa). The polyester elastomer was obtained once the Weissenberg effect was observed. The PTE with an LA molar ratio of 10% was donated as PTE-10.

**Figure 1:**
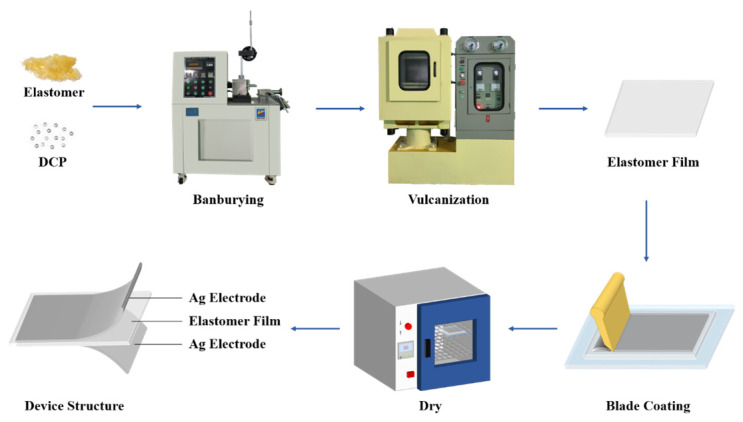
The fabrication process of the PTE sensor.

Then, 0.5 wt‰ of DCP was incorporated into the PTE under mechanically mixing at 50 °C for 30 min using the HT-TM005L Banbury Mixer (Guangdong, China). The mixture was then placed into a 2 × 2 × 0.05 cm mold and heated at 160 °C and 10 MPa pressure for 10 min using a Daylight press (XLB-D350 × 350, Shenzhen, China). Then, the cross-linked PTE was coated with a silver film on both sides to serve as the top and bottom electrodes to form the PTE sensor. The fabrication process of the PTE sensor is provided in Figure 1.

### 2.3. Characterization

The ^1^HNMR spectra were recorded using a Bruker AVANCE III HD 600 MHz spectrometer (Milwaukee, WI, USA) with CDCl3 as the solvent. The FTIR spectra were obtained with a Nicolet iS5 spectrometer (Thermo Fisher Scientific, Waltham, MA, USA). The X-ray diffraction (XRD) patterns of the samples were measured using a Rigaku Ultima IV diffractometer (Osaka, Japan). The thermal stability was evaluated by thermogravimetric analysis (TGA) on a Mettler TGA2 (Greifensee, Switzerland). The differential scanning calorimetry (DSC) was performed using a DSC 214 Polyma (NETZSCH Analyzing & Testing, Selb, Germany). The samples were heated to 200 °C under a nitrogen atmosphere at a rate of 10 °C/min and then maintained at 200 °C for 5 min; the samples were then cooled to −100 °C at 10 °C/min and finally re-heated to 200 °C. The mechanical performances of PTE were assessed using a C43304Y microcomputer-controlled (Shenzhen, China) universal testing machine.

## 3. Results and Discussions

### 3.1. Material Characterization

**Figure 2:**
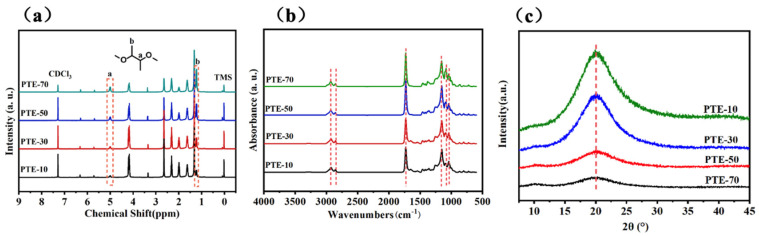
(**a**) HNMR, (**b**) FTIR, (**c**) and XRD patterns of PTE.

The ^1^HNMR spectra of PTE with different BDO contents are provided in Figure 2a. All the proton signals in PTE are detected at their expected chemical shifts, corresponding to the respective peak values. The signals for the methylene protons in the sebacic acid (–(CH_2_)_4_–CH_2_–CH_2_–COO– group) are observed at δ1.32, δ1.62, and δ2.32 ppm. The peak at δ2.64 ppm corresponds to the proton absorption of the succinic acid (–COO–(CH_2_)_2_– group). The peaks at δ1.98 and δ4.17 ppm confirm the presence of 1,3-propanediol (–(CH_2_)–CH_2_–O– group). The C=C double bonds and methylene protons of itaconic acid (–CO–C(=CH_2_)–CH_2_– group) correspond to the peaks at δ6.35, δ5.73, and δ3.36 ppm. The peaks at δ1.22 and δ5.01 ppm correspond to the methyl and methylene groups of 2,3-butanediol (–O–CH(CH_3_)– group). These two peaks indicate that the 2,3-BDO monomer has been successfully incorporated into the PTE backbone. The relative intensity of the peaks corresponding to the 2,3-BDO segments increases with the increase in 2,3-BDO content, suggesting that the actual 2,3-BDO content in the synthesized elastomer increases with the initial feed amount of 2,3-BDO.

Figure 2b presents the FTIR spectra of PTE. The absorption peaks at 2931 cm^−1^ and 2848 cm^−1^ are attributed to the symmetric and asymmetric stretching vibrations of the methylene (–CH_2_) group, respectively. The signal at 808 cm^−1^ corresponds to the stretching and bending vibrations of the –C=C–H group. The strong absorption peak at 1727 cm^−1^ is associated with the stretching vibration of the carbonyl (C=O) group, indicating the formation of abundant ester bonds. The absorption peak at 1153 cm^−1^ confirms the presence of the C–O–C=O group. The characteristic absorption peak detected at 1092 cm^−1^ corresponds to the C–O group of 2,3-butanediol, and the relative intensity of this peak increases significantly with the increase in 2,3-butanediol content, which is consistent with the 1H-NMR results.

The XRD pattern is provided in Figure 2c. A sharp diffraction peak appears at 20.12°, and as the molar ratio of 2,3-butanediol increases, the relative intensity of the diffraction peak gradually decreases until it disappears. This indicates that PTE gradually transitions from a semi-crystalline to an amorphous state. When the content of 2,3-butanediol is between 50 mol% and 70 mol%, PTE exhibits an amorphous state.

**Figure 3:**
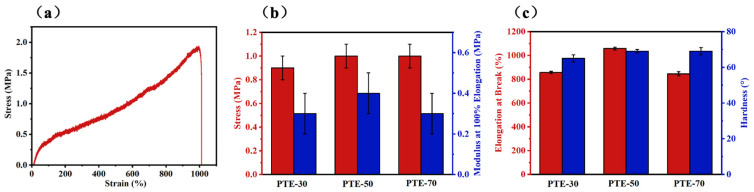
Mechanical performances of PTE: (**a**) stress–strain curves, (**b**) tensile strength and modulus at 100% elongation, (**c**) elongation at break and hardness.

The mechanical properties of PTE are shown in Figure 3. Figure 3a shows the typic stress–strain curves of PTE with elastic deformation behavior. Figure 3b,c present various mechanical parameters of PTE, including the tensile strength, modulus at 100% elongation, elongation at break, and hardness. As the content of 2,3-butanediol increases, the elastic modulus of PTE decreases, while the elongation at break of PTE initially increases and then decreases. The introduction of flexible segments such as SeA, SuA, and PDO into PTE enhances the flexibility of the macromolecular segments, forming long chains of PDO/SeA/SuA with good mobility. This leads to a reduction in the Tg of PTE, a significant decrease in the elastic modulus, and a high-elastic state at room temperature. Meanwhile, the crosslinking sites provided by IA form a stable three-dimensional network structure, while the random copolymerization of multiple monomers disrupts the ordered arrangement of macromolecular chains, reducing the regularity of the molecular segments. These factors work together to effectively suppress the crystallization of PTE, further leading to a significant decrease in its elastic modulus. As the content of 2,3-butanediol increases, PTE gradually transitions from plastic deformation to elastic deformation. However, the 2,3-butanediol content being too high causes PTE to have a decreased molecular weight, leading to poorer film-forming properties and consequently a reduced mechanical performance.

Considering the performance of PTE with different contents of 2,3-BDO, BBPE-50 exhibited the optimal mechanical properties. Therefore, BBPE-50 was selected for subsequent experimental and application studies.

### 3.2. Sensor Characterization

**Figure 4:**
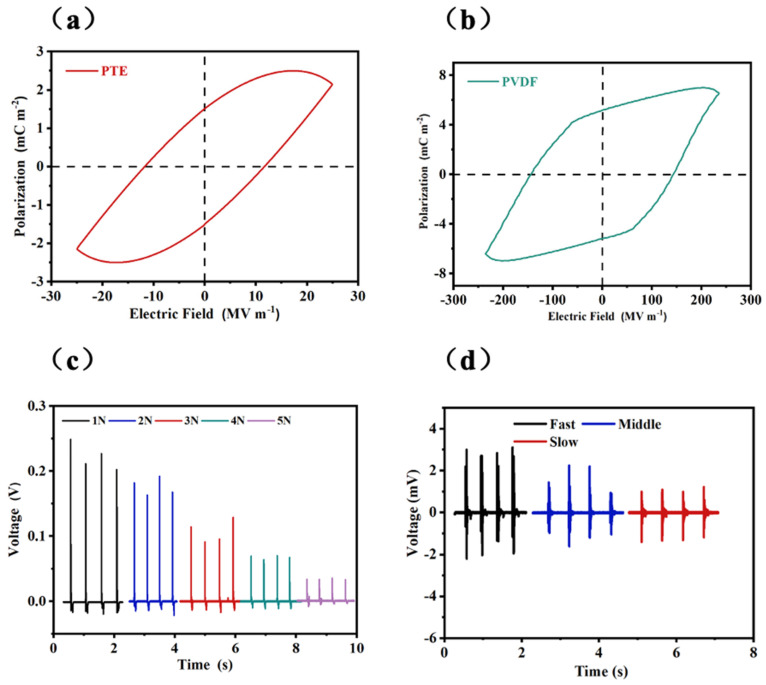
(**a**) Ferroelectric loop of PTE, (**b**) ferroelectric loop of PVDF, (**c**) piezoelectric performances of PTE, (**d**) triboelectric performances of PTE.

To investigate the piezoelectric properties of PTE, commercial PVDF piezoelectric film was used for a control group, and ferroelectric loops were performed, with the results shown in Figure 4a,b. The P-E curve was obtained at 2 kV, with a thickness of 80 µm for PTE and 8.5 µm for PVDF. PVDF shows a higher polarization electric field, 240 MV/m, and a larger Pr value, 7.3 mC/m^2^, than those of PTE. In comparison, due to the potential presence of significant current leakage, PTE exhibits an elliptical P-E curve. Under a polarization electric field of 13 MV/m, a Pr value of 1.6 mC/m^2^ was measured, indicating its obvious piezoelectricity.

The piezoelectricity of PTE is further investigated by its piezoelectric voltage responding to mechanical stimuli. The PTE sensor is applied with different mechanical vertical stress, and its output voltage is recorded in Figure 4c. As the applied stress increases, the PTE sensor shows an increasing piezoelectric voltage. With the increase in the value of the applied stress, the impact on the PTE sensor also increases, leading to large deformation in the PTE, which in turn generates more induced charge and outputs a higher piezoelectric voltage. In addition, the PTE sensor shows a different piezoelectric response under variated strain. In Figure S2, with the increase in the strain of the PTE sensor, its piezoelectric output decreases accordingly. This is because the PTE sensor shows a decreased deformation capacity under high strain, which leads to a decreased piezoelectric output.

As shown in Figure 4d, the triboelectric performance of the PTE sensor was investigated by performing a pendulum motion using a metal ball carrying a 50 × 50 cm^2^ PE film, swinging at a certain height above the PTE sensor. Each time the pendulum moved over the PTE sensor, a pulse triboelectric voltage was observed. This is because the principle of triboelectricity arises from the different electronegativity of two materials, which causes charge transfer when they interact, resulting in a potential difference that leads to triboelectric charges. Therefore, when the PE film moves above the PTE sensor, the electrostatic field strength around the PTE sensor changes, causing internal dipole variations. This leads to the accumulation of induced charge on the surface area of the sensor electrodes, generating a triboelectric voltage signal. As the moving speed between the PE film and the PTE sensor increases, the triboelectric voltage increases accordingly.

### 3.3. Contact and Contactless Response Performances

**Figure 5:**
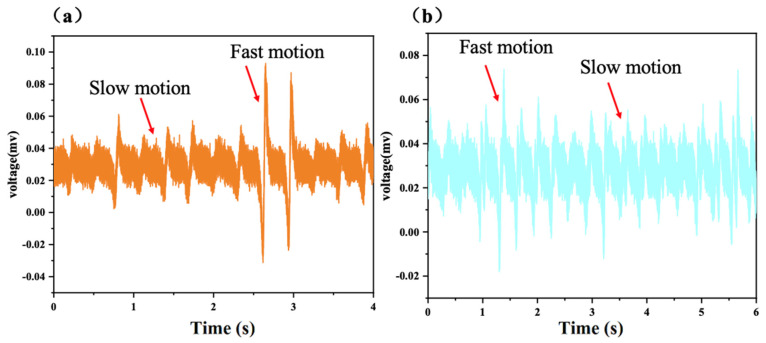
Triboelectric response for (**a**) horizontal motion and (**b**) vertical motion of PTE.

The PTE sensor is further used as the integrated sensor for contact and contactless response to mechanical stimuli. In Figure 5a,b, when the human hand moves horizontally and vertically above the PTE sensor, it can respond to each motion of the human hand by generating triboelectric charge. Meanwhile, with the motion speed increase, the PTE sensor generates increased piezoelectric voltage, indicating its capacity to distinguish the motion speed of contactless mechanical stimuli.

**Figure 6:**
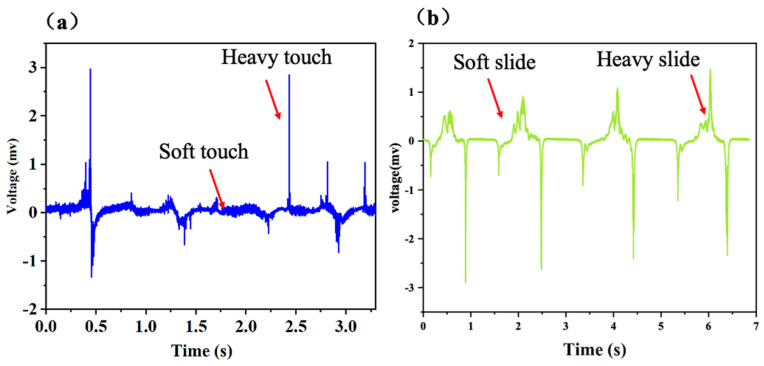
Piezoelectric response to (**a**) hand touch (**b**) finger bending of PTE sensors.

The experimental setup for the piezoelectric and triboelectric response of the PTE sensor is provided in Figure S1. The PTE sensor can also respond to contact mechanical stimuli by its piezoelectric function. As shown in Figure 6a, when a human hand presses on the PTE sensor, the sensor generates an instantaneous piezoelectric pulse peak, with a response time less than 20 ms, indicating that the PTE sensor has a high response speed. At the same time, the pulse voltage increases with the pressing stress. Similarly, when a human hand slides onto the PTE sensor (as provided in Figure 6b), a corresponding piezoelectric signal is generated, and this signal also increases with the intensity of the sliding stress, demonstrating that the PTE sensor responds well to both contact and sliding motion. In future research, by dividing the bottom electrode of the PTE sensor into regions, it will be possible to recognize the direction of contacted gesture motion. The performance of the PTE and the reported work in the reference is compared in Table S1 [22,23,24,25,26,27,28,29], and the PTE sensor shows advances in the integration of piezoelectric and triboelectric responses.

**Figure 7:**
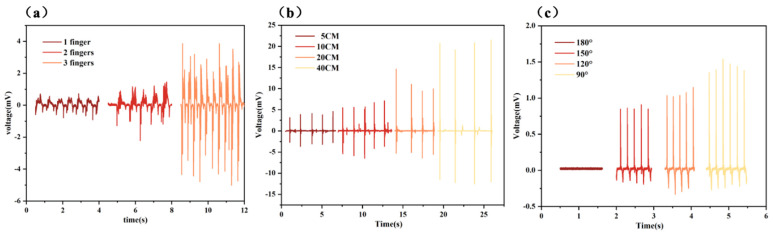
Piezoelectric output of PTE under (**a**) horizontal and (**b**) vertical motion and (**c**) finger bending condition.

Figure 7a,b show the piezoelectric response of the PTE sensor under horizontal and vertical stress, respectively. In Figure 7a, as a different number of fingers slide horizontally across the surface of the PTE sensor, its piezoelectric output signal increases with the number of fingers. This is because a larger contact area leads to a greater accumulation of piezoelectric charges. Figure 7b displays the piezoelectric response of the PTE sensor when an insulated ceramic ball falls freely from different heights onto its surface. As the falling height of the ball increases, the piezoelectric output of the PTE sensor increases accordingly. This is due to the greater deformation of the PTE sensor, which results in more accumulated charge. Figure 7c shows the output of the PTE sensor as a finger bends. As the bending angle increases, the deformation of the PTE sensor increases, leading to a higher output.

**Figure 8:**
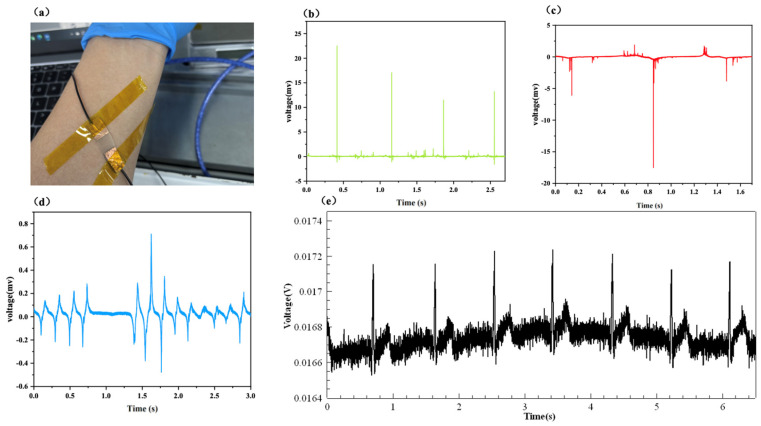
(**a**) Optical image of PTE sensor and responding voltage of PTE under (**b**) wrist bending, (**c**) hand touch, (**d**) contactless hand motion, (**e**) heartbeat.

Furthermore, the PTE sensor is attached to human skin to verify its response to both contact and non-contact mechanical stimuli. As shown in Figure 8a, the PTE sensor is attached to the wrist using adhesive tape. Figure 8b,c display the piezoelectric response voltage of the PTE sensor during wrist bending and finger pressing under wearing conditions. The PTE sensor can respond to each mechanical stimulus (including bending or pressing), and the intensity of the response voltage varies with the bending degree or pressing strength, indicating that the PTE sensor can accurately respond to external contact-based mechanical stimuli while in the wearing state. As shown in Figure 8d, when the human hand slides over without any contact with human skin, the PTE sensor can also respond to each slide motion, generating corresponding triboelectric voltage peaks. Additionally, the peak voltage of the triboelectric voltage increases with the motion speed. As shown in Figure 8e, we also attempted to use the PTE sensor in heartbeat detection. The PTE sensor produced corresponding piezoelectric voltage peaks in response to the human heartbeat pulse. By calculating the time corresponding to the width of the piezoelectric pulse peaks and the number of peaks, the wearer’s heart rate is estimated to be 85 beats per minute, which is in close agreement with the measured value of 84 beats per minute. The PTE sensor shows a constant piezoelectric output after 4000 impact cycles, as shown in Figure S3, which shows good durability.

## 4. Conclusions

Human–computer interaction (HCI) enables communication between humans and machines, and the touch control and gesture recognition methods provided high precision and convenient experience. In the present work, a polyester elastomer (PTE) with both piezoelectric and triboelectric properties was successfully synthesized through the copolymerization of various diols and diacids. The incorporation of long carbon chains in the polymer backbone provides molecular mobility, while the random copolymerization and crosslinking effectively suppress crystallization, ensuring its stretchability. The high density of C=O dipoles in the ester bonds enables excellent piezoelectric and triboelectric performance without requiring polarization. As a result, the stretchable PTE sensor is capable of simultaneously achieving both piezoelectric and triboelectric responses to contacted and contactless mechanical stimuli. Meanwhile, the PTE sensor can respond synchronously and accurately to slide, touch, and hand motion in the wearing state, showing great potential in HCI fields.

## Data Availability

The original contributions presented in this study are included in the article/Supplementary Materials. Further inquiries can be directed to the corresponding author.

## Supplementary Materials

The following supporting information can be downloaded at: https://www.mdpi.com/article/10.3390/polym17081105/s1, Figure S1: Experimental setup of piezoelectric and triboelectric response of PTE sensor. Table S1: performances comparation of PTE sensor and related work. Figure S2: Piezoelectric performances of PTE sensor under different strain. Figure S3: Piezoelectric output of PTE sensor after 4000 impact cycles. References [22,23,24,25,26,27,28,29] are cited in the Supplementary Materials.
