# Supplementary material for: Stretchable and Wearable Sensors for Contact Touch and Gesture Recognition Based on Poling-Free Piezoelectric Polyester Elastomer
Source: Polymers (Basel). 2025 Apr 19;17(8):1105. doi: 10.3390/polym17081105 (PMC12030288; doi:10.3390/polym17081105)
Supplement: Supplementary file 1 [file polymers-17-01105-s001.zip › polymers-3544128-supplementary.pdf]

**Stretchable and wearable sensors for contact touch and gesture recognition based on poling-free piezoelectric polyester elastomer**

**Supporting Information**

*Kaituo Wu[a], Wanli Zhang[a], Qian Zhang[a,*], and Xiaoran Hu[*,b]*

[a] State Key Laboratory of Electronic Thin Films and Integrated Devices, University of Electronic Science and Technology of China, Chengdu 611731, China

[b] School of Materials and Energy, University of Electronic Science and Technology of China, Chengdu, China

(Correspondence to Xiaoran Hu, e-mail: Huxiaoran@uestc.edu.cn)

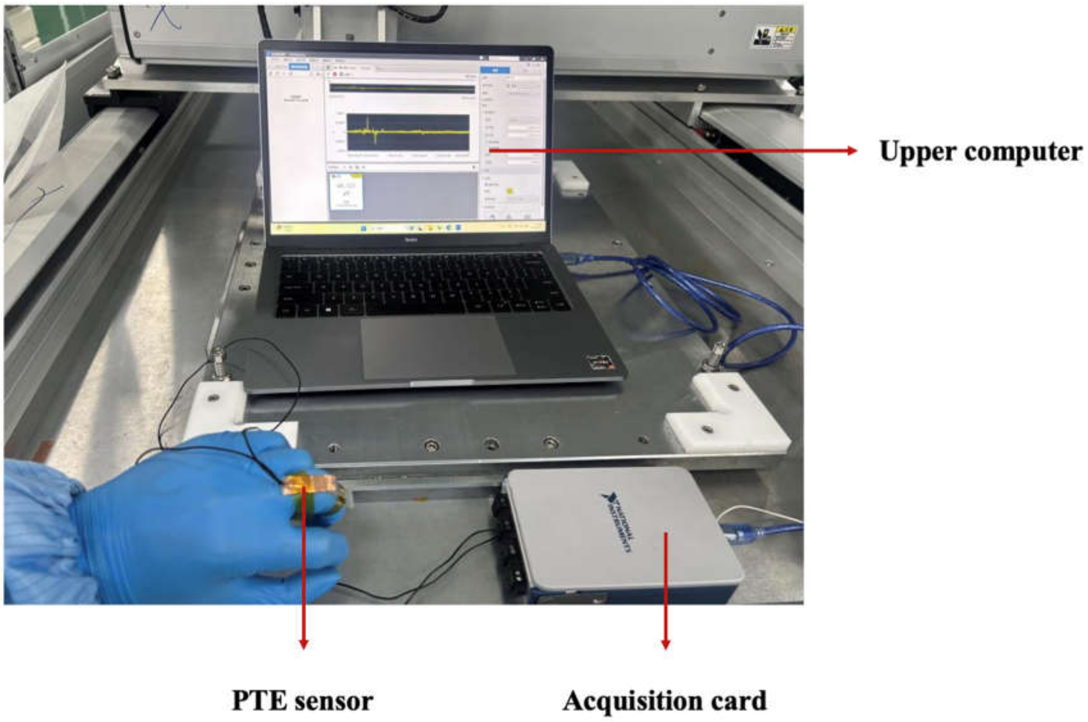


**Figure S1** Experimental setup of piezoelectric and triboelectric response of PTE sensor

Table S1 performances comparation of PTE sensor and related work

| Work | Tensile properties | sensing mode | piezoelectric coefficient($d_{33}$) | Response time |
|---|---|---|---|---|
| PVDF-PAN(1) | 259.3% | piezoelectricity | 63pC/N | 15ms |
| MXene/Ag(2) | - | piezoelectricity | - | 100ms |
| PUVE（3） | - | piezoelectricity | - | 40ms |
| P（NaSS-co-AN）（4） | 250% | piezoelectricity | 40pC/N | - |
| Double-sandwich structure triboelectric sensor(5) | - | triboelectricity | - | 26ms |
| PAN/SA(6) | - | triboelectricity | - | 7ms |
| P-TPS（7） | ＜200% | Triboelectricity piezoelectricity | - | 8.8ms |
| PME(8) | - | piezoelectricity | - | 55ms |
| PTE | ＞1000% | Triboelectricity piezoelectricity | | 15ms |

[1] Guan Y , Tu L , Ren K ,et al.Soft, Super-Elastic, All-Polymer Piezoelectric Elastomer for Artificial Electronic Skin[J].[2025-04-07].
[2] Li L , Pan J , Chang L ,et al.A MXene heterostructure-based piezoionic sensor for wearable sensing applications[J].Chemical Engineering Journal, 2024, 482.DOI:10.1016/j.cej.2024.148988.
[3] Wang F , Yang P , Liu W ,et al.Simultaneous Visualization of Dynamical and Static Tactile Perception Using Piezoelectric-Ultrasonic Bimodal Electronic Skin Based on In Situ Polarized PVDF-TrFE/2DBP Composites and the TFT Array[J].
[4] Fu R , Tu L ,Guan, YoujunWang, ZhengaoDeng, ChunlinYu, PengTan, GuoxinNing, ChengyunZhou, Lei.Intrinsically piezoelectric elastomer based on crosslinked polyacrylonitrile for soft electronics[J].Nano Energy, 2022, 103(Pt.A).DOI:10.1016/j.nanoen.2022.107784.
[5] Xu R , Luo F , Zhu Z ,et al.Flexible Wide-Range Triboelectric Sensor for Physiological Signal Monitoring and Human Motion Recognition[J].ACS Applied Electronic Materials, 2022, 4(8):10.DOI:10.1021/acsaelm.2c00681.

[6] Hong K , Hao Y , Yang J ,et al.Sodium–Alginate Composite Nanofiber-Based Triboelectric Sensor for Self-Powered Wrist Posture Identification[J].ACS APPLIED ELECTRONIC MATERIALS, 2024, 6(12):9071-9081.DOI:10.1021/acsaelm.4c01722.
[7] Yu J , Chen L , Hou X ,et al.Stretchable and skin-conformal piezo-triboelectric pressure sensor for human joint bending motion monitoring - ScienceDirect[J].Journal of Materiomics, 2022.
[8] Yang T , Pan H , Tian G ,et al.Hierarchically Structured PVDF/ZnO Core-shell Nanofibers for Self-powered Physiological Monitoring Electronics[J].Nano Energy, 2020, 72:104706.DOI:10.1016/j.nanoen.2020.104706.

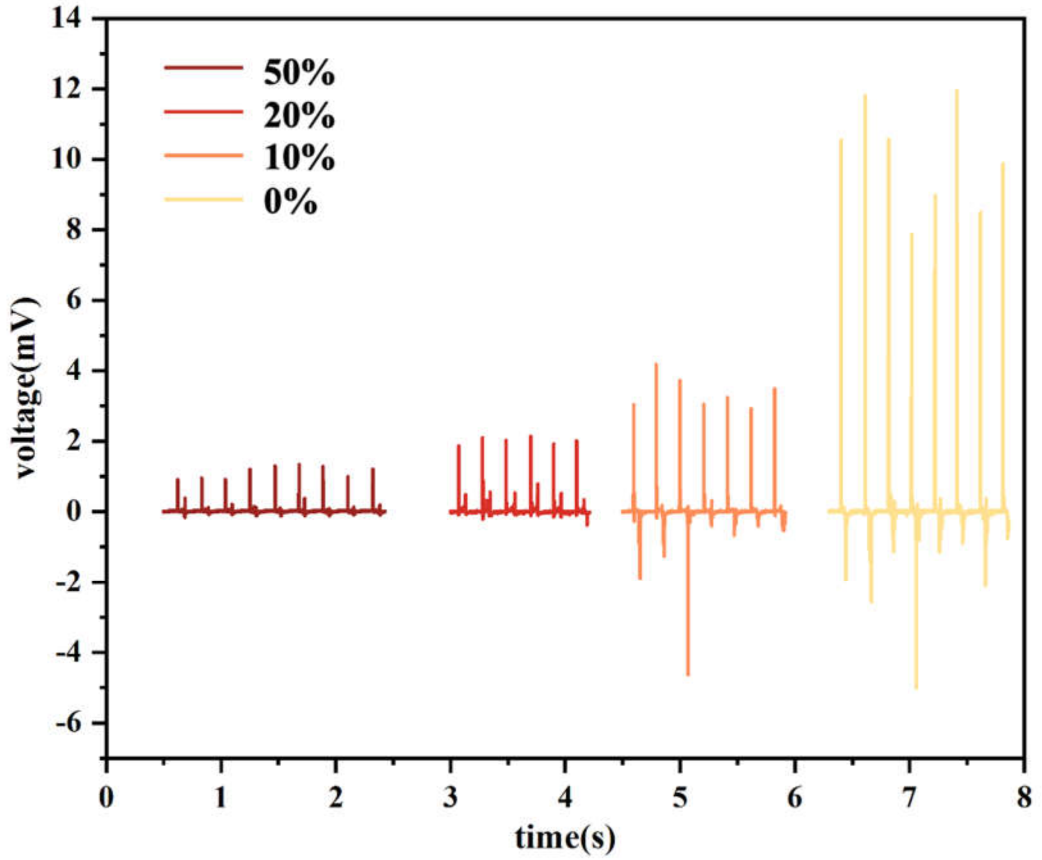


Figure S2 Piezoelectric performances of PTE sensor under different strain

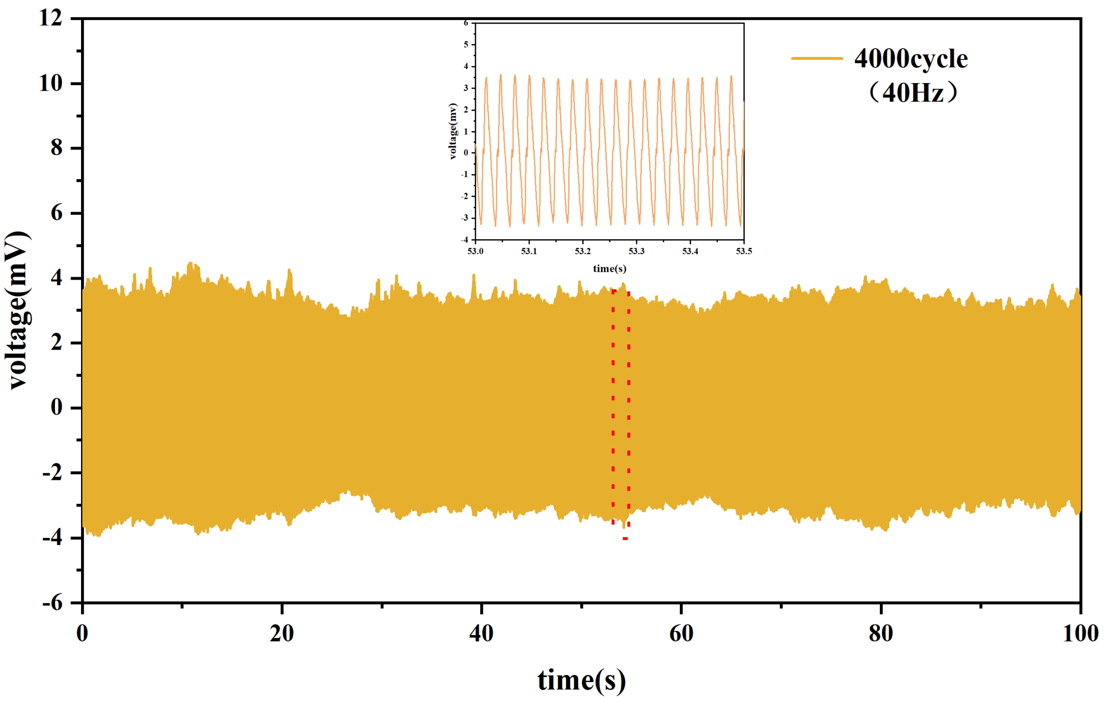


Figure S3 Piezoelectric output of PTE sensor after 4000 impact cycles
